# Supplementary material for: Comparison between Multiple Doses and Single-Dose Steroids in Preventing the Incidence of Reintubation after Extubation among Critically Ill Patients: A Network Meta-Analysis
Source: J Clin Med. 2021 Jun 29;10(13):2900. doi: 10.3390/jcm10132900 (PMC8268958; doi:10.3390/jcm10132900)

Supplementary materials

Table S1. GRADE of each comparison

Table S2. Search strategy

Table S3. Glucocorticoid equivalents

Figure S1. Risk of bias of included studies

Figure S2. Network map

Figure S3. Forest plot of each comparison

Figure S4. The inconsistency test at the global and local levels.

Figure S5. Funnel plot of included studies

Table S1. GRADE of each comparison

Multiple doses compared to Placebo for Reintubation

Bibliography:

Certainty assessment							Summary of findings				
Participants (studies) Follow up	Risk of bias	Inconsistency	Indirectness	Imprecision	Publication bias	Overall certainty of evidence	Study event rates (%)		Relative effect (95% CI)	Anticipated absolute effects	
							With Placebo	With Multiple doses		Risk with Placebo	Risk difference with Multiple doses
**Reintubation_ Multiple doses vs Placebo**											
1191 (6 RCTs)	not serious	not serious	not serious	not serious	none	⨁⨁⨁⨁ HIGH	49/568 (8.6%)	23/623 (3.7%)	**OR 0.43** (0.25 to 0.72)	86 per 1,000	**47 fewer per 1,000** (from 63 fewer to 23 fewer)
**Reintubation_ Single dose vs Placebo**											
1003 (5 RCTs)	not serious	not serious	not serious	not serious	none	⨁⨁⨁⨁ HIGH	26/504 (5.2%)	9/499 (1.8%)	**OR 0.31** (0.14 to 0.69)	52 per 1,000	**35 fewer per 1,000** (from 44 fewer to 15 fewer)
**Reintubation_ Multiple doses vs Single dose**											
194 (2 RCTs)	not serious	not serious	not serious	serious	none	⨁⨁⨁◯ MODERATE	4/95 (4.2%)	5/99 (5.1%)	**OR 1.22** (0.32 to 4.74)	42 per 1,000	**9 more per 1,000** (from 28 fewer to 130 more)

Table S2. Search strategy

Database	Search	Studies
Medline	#1. Intubation [MeSH Terms] OR Intubation, Intratracheal [MeSH Terms] OR intubation.ti,ab,kw (74,313)<br>#2. Airway Extubation [MeSH Terms] OR extubation.ti,ab,kw (9,774)<br>#3. Airway Obstruction [MeSH Terms] OR airway obstruction (32,349)<br>#4. stridor*.ti,ab,kw (3,778)<br>#5. (laryn*.ti,ab,kw AND edema*.ti,ab,kw) OR laryngeal edema.ti,ab,kw (1,777)<br>#6. or/1-5 (112,118)<br>#7. Adrenal Cortex Hormones [MeSH Terms] OR Steroids [MeSH Terms] Glucocorticoids [MeSH Terms] OR steroid*.ti,ab,kw OR corticosteroid*.ti,ab,kw OR glucocorticoid*.ti,ab,kw (1,060,038)<br>#8. Prednisone [MeSH Terms] OR prednisone*.ti,ab,kw (51,185)<br>#9. Prednisolone [MeSH Terms] OR prednisolone*.ti,ab,kw (60,562)<br>#10. Methylprednisolone [MeSH Terms] OR methylprednisolone*.ti,ab,kw (25,032)<br>#11. Dexamethasone [MeSH Terms] OR dexamethasone*.ti,ab,kw (68,065)<br>#12. Cortisone [MeSH Terms] OR cortisone*.ti,ab,kw (23,124)<br>#13. Hydrocortisone [MeSH Terms] OR hydrocortisone*.ti,ab,kw (75,757)<br>#14. Budesonide [MeSH Terms] OR budesonide*.ti,ab,kw (5,839)<br>#15. Fluticasone [MeSH Terms] OR fluticasone*.ti,ab,kw (4,299)<br>#16. ciclesonide*.ti,ab,kw (336)<br>#17. Triamcinolone [MeSH Terms] OR triamcinolone*.ti,ab,kw (11,165)<br>#18. Beclomethasone {MeSH Terms} OR beclomethasone*.ti,ab,kw (3,743)<br>#19. flunisolide*ti,ab,kw (309)<br>#20. momethasone*.ti,ab,kw (10)<br>#21. or/7-20 (1,095,201)<br>#22. Randomized controlled trial.pt OR controlled clinical trial.pt OR randomized.ti,ab OR placebo.ti,ab OR drug therapy.sh OR randomly.ti,ab OR trial.ti,ab OR groups.ti,ab (2,797,200)<br>#23. 6 AND 21 AND 22 (1,914)	1,914
Embase	#1. Endotracheal Intubation [E] OR Intubation [E] OR intubation.ti,ab,kw (100,387)<br>#2. Extubation [E] OR extubation.ti,ab,kw (21,364)<br>#3. Airway Obstruction [E] OR airway obstruction (42,647)<br>#4. Stridor [E] OR stridor.ti,ab,kw (8,768)<br>#5. (laryn*.ti,ab,kw AND edema*.ti,ab,kw) OR laryngeal edema.ti,ab,kw (2,750)<br>#6. or/1-5 (158,209)<br>#7. Corticosteroid [E] OR Steroid [E] OR Glucocorticoid [E] OR steroid*.ti,ab,kw OR corticosteroid*.ti,ab,kw OR glucocorticoid*.ti,ab,kw (1,485,027)<br>#8. Prednisone [E] OR prednisone*.ti,ab,kw (164,062)<br>#9. Prednisolone [E] OR prednisolone*.ti,ab,kw (122,080)<br>#10. Methylprednisolone [E] OR methylprednisolone*.ti,ab,kw (90,720)<br>#11. Dexamethasone [E] OR dexamethasone*.ti,ab,kw (148.354)<br>#12. Cortisone [E] OR cortisone*.ti,ab,kw (15,302)<br>#13. Hydrocortisone [E] OR hydrocortisone*.ti,ab,kw (119,640)<br>#14. Budesonide [E] OR budesonide*.ti,ab,kw (20,529)<br>#15. Fluticasone [E] OR fluticasone*.ti,ab,kw (11,754)<br>#16. Ciclesonide [E] OR ciclesonide*.ti,ab,kw (1,484)<br>#17. Triamcinolone [E] OR triamcinolone*.ti,ab,kw (18,778)<br>#18. Beclomethasone [E] OR beclomethasone*.ti,ab,kw (9,534)<br>#19. Flunisolide [E] OR flunisolide*ti,ab,kw (2,411)<br>#20. Momethaone Furoate [E] OR momethasone*.ti,ab,kw (4,552)<br>#21. or/7-20 (589,724)<br>#22. randomized.ti,ab OR placebo.ti,ab OR drug therapy.sh OR	1,844

	randomly.ti,ab OR trial.ti,ab OR groups.ti,ab (4,051,545)<br>#23. 6 AND 21 AND 22 (1,844)	
Cochrane library	#1. Intubation [MeSH] OR Intubation,Intratracheal [MeSH] OR intubation OR Airway Extubation [MeSH] OR extubation OR Airway Obstruction [MeSH] OR airway obastruction OR stridor OR laryngeal edema (18,732)<br><br>#2. Adrenal Cortex Hormones [MeSH] OR Steroids [MeSH] OR Glucocorticoids [MeSH] OR steroid OR corticosteroid OR glucocorticoid OR prednisone OR prednisolone OR methylprednisolone OR dexamethasone OR cortisone OR hydrocortisone OR budesonide OR fluticasone OR ciclesonide OR triamcinolone OR beclomethasone OR flunisolide OR mometasone (88,274)<br><br>#3. 1 AND 2 (1,871)	1,871
Total		5,629

Table S3. Glucocorticoid equivalents

Glucocorticoid	Dose equivalent
**Hydrocortisone**	**100 mg**
Prednisone	25 mg
Prednisolone	25 mg
Triamcinolone	20 mg
Methylprednisolone	20 mg
Betamethasone	4 mg
Dexamethasone	4 mg

Figure S1. Risk of bias of included studies

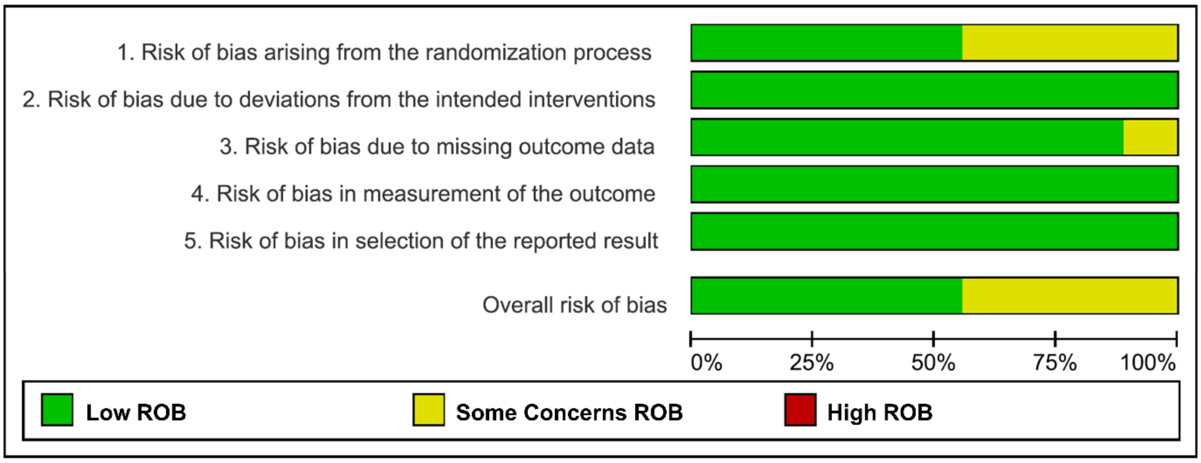

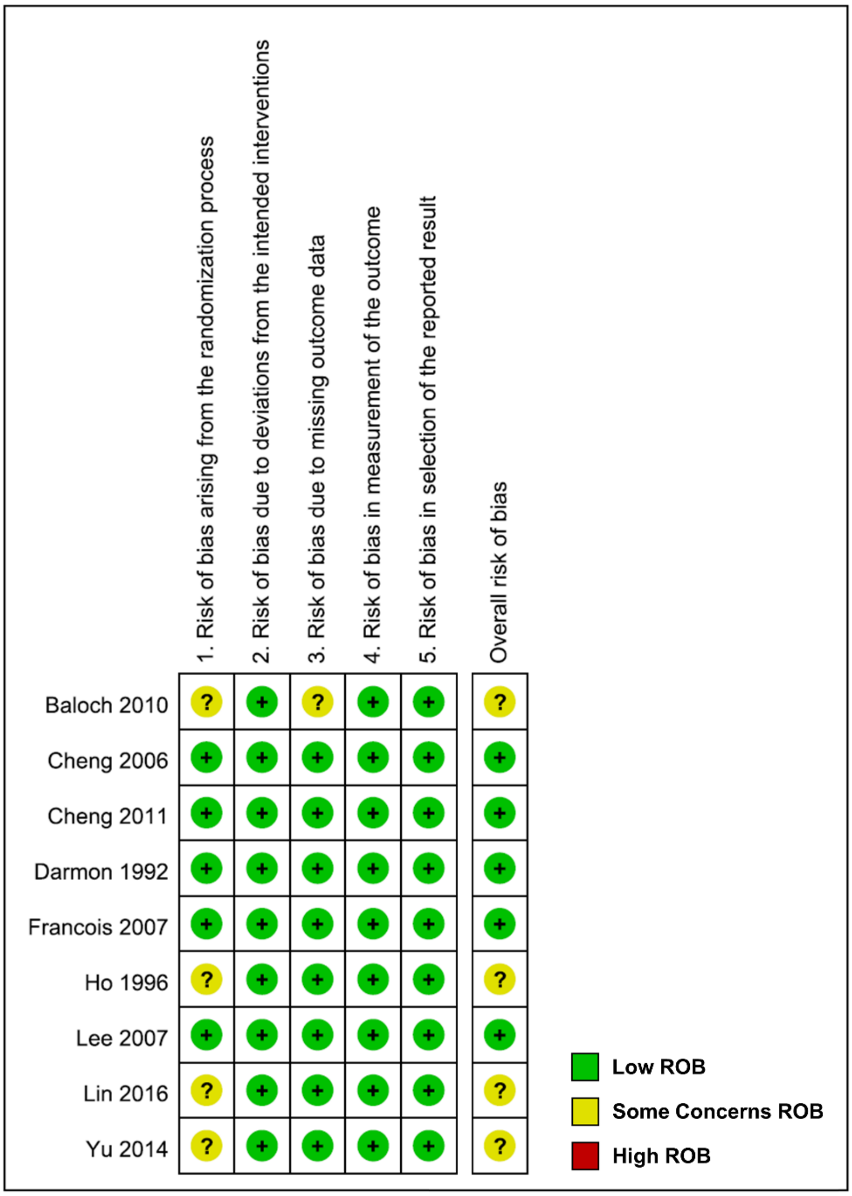

Figure S2. Network map

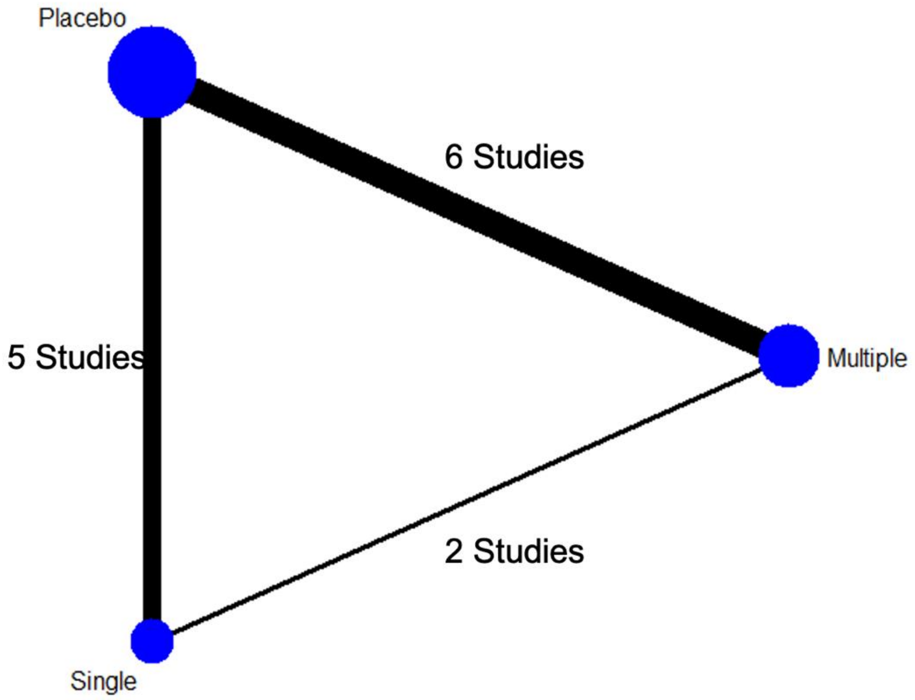

Figure S3. Forest plot of each comparison

1. Multiple doses vs Placebo

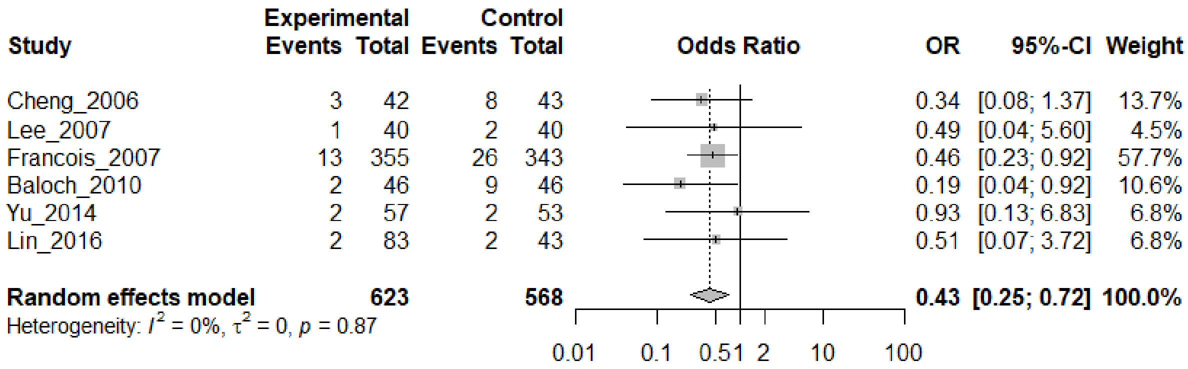

2. Single dose vs Placebo

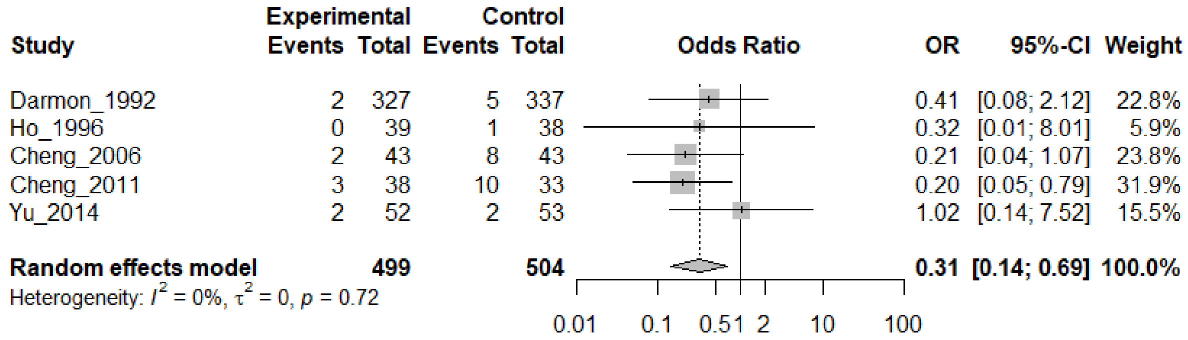

3. Multiple doses vs Single dose

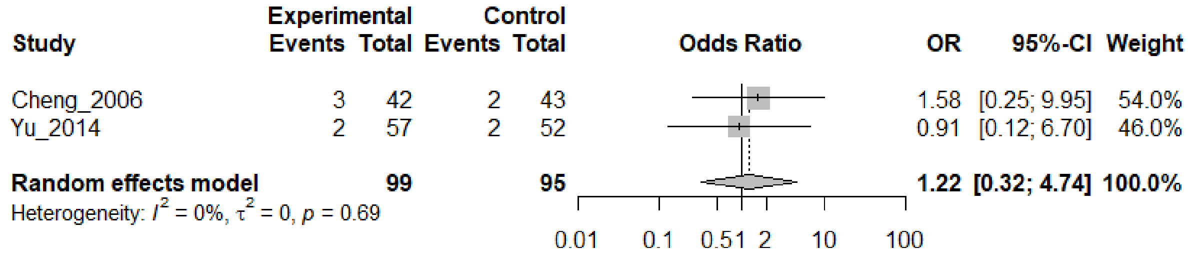

Figure S4. The inconsistency test at the global and local levels.

1. Global approach

```
3 . network meta i, eform
  Command is: mvmeta _y _S  , eform  bscovariance(exch 0.5) longparm suppress(uv mm
  > ) eq(_y_A: des_ABC, _y_C: des_BC) vars(_y_A _y_C)
  Note: using method  reml
  Note: regressing  _y_A on des_ABC
  Note: regressing  _y_C on des_BC
  Note: 9 observations on  2 variables
  Note: variance-covariance matrix is   proportional to .5*I(2)+.5*J(2,2,1)

  initial:       log likelihood =   -11.141144
  rescale:       log likelihood =   -11.141144
  rescale eq:    log likelihood =   -9.3084664
  Iteration 0:   log likelihood =   -9.3084664
  Iteration 1:   log likelihood =   -9.2256785
  Iteration 2:   log likelihood =   -9.2256784

  Multivariate meta-analysis
  Variance-covariance matrix =   proportional .5*I(2)+.5*J(2,2,1)
  Method = reml                                 Number of dimensions     =        2
  Restricted log likelihood =    -9.2256784     Number of observations   =        9
```

	exp(Coef)	Std. Err.	z	P>|z|	[95% Conf. Interval]	
**_y_A**						
des_ABC	1.074003	.7024869	0.11	0.913	.2980225	3.870453
_cons	.4148779	.1231193	-2.96	0.003	.2319089	.7422038
**_y_C**						
des_BC	.7108566	.58027	-0.42	0.676	.143532	3.520587
_cons	.3821459	.2418653	-1.52	0.129	.1105331	1.321193

```
  Estimated between-studies SDs and correlation matrix:
                SD        _y_A       _y_C
  _y_A   7.964e-12           1          .
  _y_C   7.964e-12          .5          1

  Testing for inconsistency:
   ( 1)   [_y_A]des_ABC = 0
   ( 2)   [_y_C]des_BC = 0

             chi2(  2) =      0.17
           Prob > chi2 =      0.9163
  mvmeta command stored as F9; test command stored as F8
```

2. Local approach

```
7 . network sidesplit all
```

Side	Direct Coef.	Std. Err.	Indirect Coef.	Std. Err.	Difference Coef.	Std. Err.	P>|z|
B C *	-1.167121	.3997071	-1.174295	1.246857	.0071746	1.267316	0.995
A B *	.865075	.2644586	1.50346	1.569214	-.6383855	1.581613	0.686
A C	-.1720447	.687635	-.3695049	.5749717	.1974602	.8874279	0.824

```
  * Warning: all the evidence about these contrasts comes from the trials which dir
  > ectly compare them.
  See help file  for more information.
```

Figure S5. Funnel plot of included studies. A, placebo; B, multiple doses; C, single dose.

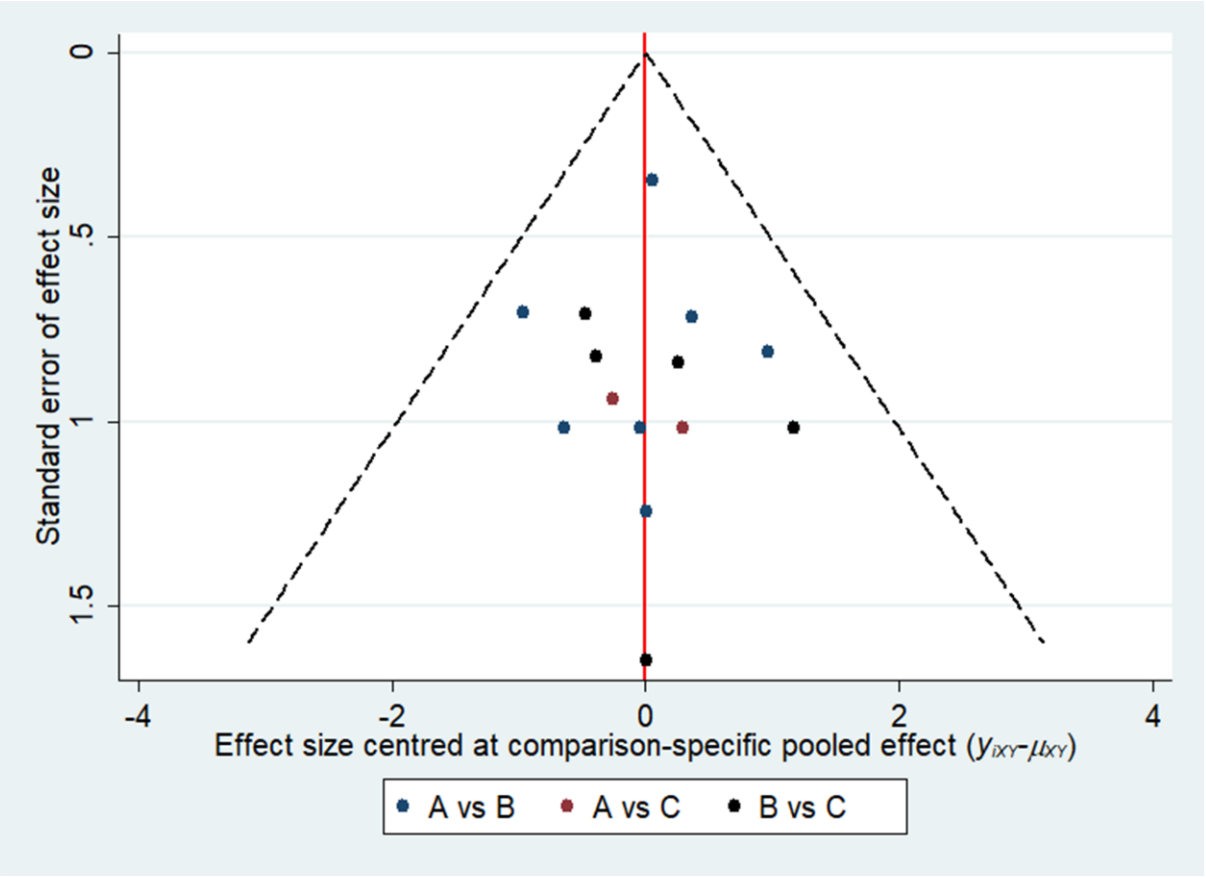

Supplement: Supplementary file 1 [file jcm-10-02900-s001.zip › jcm-1272233-supplementary.pdf]
